# Predicting adverse pregnancy outcomes of pregnant mothers with syphilis based on a logistic regression model: a retrospective study

**DOI:** 10.3389/fpubh.2023.1201162

**Published:** 2023-09-14

**Authors:** Yu-Wei Zhang, Man-Yu Liu, Xing-Hao Yu, Xiu-Yu He, Wei Song, Xiao Liu, Ya-Na Ma

**Affiliations:** ^1^Department of Child and Adolescent Health and Social Medicine, School of Public Health, Medical College of Soochow University, Suzhou, China; ^2^Suzhou Maternal and Child Health Care Family Planning Service Center, Suzhou, China; ^3^Department of Science and Education, The Affiliated Infectious Diseases Hospital of Soochow University, The Fifth People's Hospital of Suzhou, Suzhou, China

**Keywords:** maternal syphilis, adverse pregnancy outcomes, APOs, nomogram, logistic (logit) regression

## Abstract

**Objective:**

Maternal syphilis could cause serious consequences. The aim of this study was to identify risk factors for maternal syphilis in order to predict an individual's risk of developing adverse pregnancy outcomes (APOs).

**Methods:**

A retrospective study was conducted on 768 pregnant women with syphilis. A questionnaire was completed and data analyzed. The data was divided into a training set and a testing set. Using logistic regression to establish predictive models in the training set, and its predictive performance was evaluated in the testing set. The probability of APOs occurrence is presented through a nomogram.

**Results:**

Compared with the APOs group, pregnant women in the non-APOs group participated in a longer treatment course. Course, time of the first antenatal care, gestation week at syphilis diagnosis, and gestation age at delivery in weeks were independent predictors of APOs, and they were used to establish the nomogram.

**Conclusions:**

Our study investigated the impact of various characteristics of syphilis pregnant women on pregnancy outcomes and established a prediction model of APOs in Suzhou. The incidence of APOs can be reduced by controlling for these risk factors.

## 1. Introduction

Syphilis is a sexually transmitted infection caused by the spirochete *Treponema pallidum* that increases the risk of HIV infection in addition to directly contributing to morbidity and mortality. *Treponema pallidum* invading brain parenchyma can cause nerve cell degeneration, tissue atrophy, frontal lobe lesions. It also can infect infants through vertical transmission and may present with lifelong diseases, including irreversible neurological and cardiovascular complications (1–3). Most maternal syphilis infections are latent, and mothers with syphilis often do not have awareness of it until they are diagnosed (4). Previous studies have concluded that untreated infections can lead to adverse pregnancy outcomes (APOs) (5). Historical and present studies suggest that untreated syphilis or incompletely treated in pregnancy carries a significant risk of stillbirth, prematurity or low birth weight, neonatal death, and congenital syphilis, among others (6–9). It was estimated that the global maternal syphilis prevalence was 0.69% and the probability of APOs occurrence as a regional median of 6.8% (range, 3.7%–10.4%) for the African region and 4.4% (2.7%–6.8%) for the Americas region, for the Eastern Mediterranean region it was 5.5% (3.5%–12.5%), for the European region, 3.4% (2.3%–6.0%), for the South-East Asia region, 6.0% (2.9%–7.9%), and for the Western Pacific region, 4.7% (2.3%–12.2%), causing a substantial public health burden and cost to families (10). Located in the middle of the Yangtze River Delta, Suzhou is famous for its national high-tech industrial base. The population of Suzhou is 10.72 million, of which 50% are transient. In 2018, the total number of pregnant women and live births in Suzhou were 111,488 and 112,523, respectively (11). The city has a large floating population and migrant workers, a demographic structure that is more common in some relatively developed cities in China. Therefore, the sample from this city is representative of maternal and neonatal health in cities with similar levels of economic development.

A Portuguese study indicated that sociodemographic, behavioral, and health care factors are determinant factors for gestational syphilis (12). In addition, a study from Zhejiang Province, China, demonstrated its progress in eliminating mother-to-child transmission from 2015 to 2020: there was a significant increase in both screening coverage and treatment coverage, yet the overall incidence of APOs did not decrease significantly (13). Studies have used prediction tools to predict HIV risk in pregnant women and to prioritize pregnant women for preexposure prophylaxis (14). Current research on maternal syphilis has focused on congenital syphilis (CS) rather than APOs, and given that the existing literature confirms that the risk of developing APOs in maternal syphilis can be reduced (15–19), the aim of this study was to identify risk factors for maternal syphilis in order to predict an individual's risk of developing APOs, thereby guiding the review and avoiding the development of adverse events.

## 2. Methods

### 2.1. Participants

In this retrospective study, the multicentered clinical data of pregnant women with syphilis and their fetuses were obtained from the outpatient clinic of maternal and child health care institutions in Suzhou, Jiangsu Province, China. All syphilis-infected pregnant women with average age is 29.51 years who delivered ≥28 gestational weeks were registered between January 2018 and December 2020 and were enrolled, regardless of their birth outcomes. The Chinese government provides comprehensive syphilis intervention services for all pregnant women free of charge. These services included syphilis counseling and testing at the first antenatal care, treatment for positive cases during pregnancy, follow-up service for exposed infants until they were diagnosed or excluded CS, which described in detail in Li and Wang (13, 20). This study was approved by the Ethics committee of The Affiliated Infectious Diseases Hospital of Soochow University (No. 2019002), and has been performed in accordance with the Declaration of Helsinki. All infected mothers were required to complete a routine questionnaire upon receiving syphilis screening and testing at their first antenatal care or delivery. Informed consent was obtained from syphilis-infected mothers. Prenatal syphilis screening and testing processes followed the guidelines and regulations of the integrated National Preventing Mother to Child Transmission Program of HIV, Syphilis, and Hepatitis B. In the final database, only mothers and infant numbers were listed. All personal information was kept confidential.

### 2.2. Definitions

The adverse pregnancy outcomes involved in this study included stillbirth, preterm birth, low birth weight (LBW), congenital disabilities, asphyxia, pneumonia, and neonatal CS. Gestational age was based on the interval between the date of the last menstrual period and the date of delivery. Preterm delivery was defined as delivery before 37 completed weeks of gestation. LBW was described as a birth weight of <2,500 g. The diagnostic criteria of neonatal CS in the study referred to Dou et al. (21).

### 2.3. Data analysis

Pregnant women with APOs were used as the study group and those without APOs were used as the control group. Relationships between the fetus with APOs and without APOs were assessed with the χ^2^ test or Fisher's exact test as appropriate. We randomly divide all samples into a training set (70%) and a test set (30%) according to the random number table. The univariable screening was performed using logistic regression with an inclusion level of 0.05. Then multinomial logistics regression was performed for the statistically significant variables. And the odds ratios (ORs) and their 95% confidence intervals (CIs) of the factors associated with APOs were estimated. Nomogram, a predictive device, was constructed to predict the probability of outcomes and visualize the results. Next, we evaluated the predictive power of the constructed models in the training and test sets: receiver operating curves, calibration curves, and decision curves were used, respectively. All analyses were performed in R software (version 3.5.3), and the significance level was set to 0.05.

## 3. Results

### 3.1. Basic information on the distribution of the participants

During the observation period from January 2018 to December 2020, 786 pregnant women with syphilis were seen in the outpatient departments of hospitals within the Suzhou metropolitan area. The characteristics of pregnant women with syphilis are shown in Table 1. Their average age was 29.51 ± 5.25 years, and they received an average of 1.54 ± 0.80 courses of treatment. The median gestational age at which they were diagnosed with syphilis was 15 weeks. The majority of these women were married, accounting for 90.9% (722) of the total, with 7.3% (57) and 0.9% (7) being unmarried and divorced, respectively. Infected women of Han ethnicity accounted for 94.4% (742), and 17.1% (128) have a bachelor's degree or higher. Of these, 50.6% (398) had a history of syphilis infection, and 71.2% had a history of APOs with their child born in the past. Table 1 provides further details.

**Table 1:** Characteristics of syphilis pregnant women (*N* = 786).

	**Mean**	**SD**	**Median**	**IQR**	***N***	**%**
Course	1.54	0.80				
Age	29.51	5.25				
Time of the first antenatal care			13.00	[12.00, 16.00]		
Gestation week at syphilis diagnosis			15.00	[13.00, 22.50]		
Gestation age at delivery in weeks			39.00	[38.00, 40.00]		
TPPA titer			2.00	[1.00, 4.00]		
**Marital status**						
Unmarried					57	7.3
Married					722	91.9
Divorced					7	0.9
**Ethnicity**						
Han					742	94.4
Others					44	5.6
**Education**						
Illiterate/semi-literate					6	0.8
Primary school					48	6.4
Junior high school					565	75.6
University or college					127	17.0
Master and above					1	0.1
**History of syphilis infections**						
Yes					398	50.6
No					388	49.4
**History of APOs**						
Yes					560	71.2
No					226	28.8

Junior high school including junior high school, vocational high school, technical school, etc.

APOs, adverse pregnancy outcomes; RPR, rapid plasma regain; TRUST, toluidine red unheated serum test; TPPA, treponema pallidum particle agglutination.

### 3.2. Comparison of maternal characteristics between newborns with APOs and newborns without APOs

The characteristics of syphilis pregnant women between the newborns with APOs and newborns without APOs are shown in Table 2. Women in the non-APOs group generally participated in a longer treatment course than women in the APOs group. Significant differences in marital status (*p* = 0.003) and treponema pallidum particle agglutination (TPPA) titer (*p* = 0.03) were observed between the two groups. And more people in the non-APOs group had a history of syphilis infection than in the APOs group (*p* = 0.02). In addition, compared with women in the non-APOs group, those in the APOs group had a younger gestational age at delivery in weeks. Further details are provided in Table 2.

**Table 2:** Comparison of maternal sociodemographic characteristics and syphilis condition between newborns with APOs and newborns without APOs.

	**Without APOs 648**	**With APOs 138**	***P-*value**
	**Mean (SD)/*****M*** **(IQR)/*****N*** **(%)**	**Mean (SD)/*****M*** **(IQR)/*****N*** **(%)**	
Course	1.59 (0.78)	1.30 (0.87)	< 0.001
Age	29.51 (5.12)	29.49 (5.81)	0.96
Gravidity	3.00 [2.00, 4.00]	3.00 [2.00, 4.00]	0.72
Parity	1.00 [0.00, 1.00]	1.00 [0.00, 1.00]	0.76
Number of children present	1.00 [0.00, 1.00]	0.00 [0.00, 1.00]	0.73
Time of the first antenatal care	13.00 [12.00, 16.00]	13.00 [11.25, 23.25]	0.13
Gestation week at syphilis diagnosis	15.00 [13.00, 21.00]	16.00 [13.00, 31.00]	0.054
Gestation age at delivery in weeks	39.00 [38.00, 40.00]	37.00 [35.00, 39.00]	< 0.001
RPR/TRUST Titer	2.00 [1.00, 4.00]	2.00 [1.00, 7.00]	0.06
TPPA titer	2.00 [1.00, 4.00]	2.00 [1.00, 8.00]	0.03
RPR/TRUST titer of newborns	2.00 [1.00, 2.00]	2.00 [1.00, 6.00]	0.02
**Ethnicity**			
Han	611 (94.3)	131 (94.9)	0.93
Others	37 (5.7)	7 (5.1)	
**Education**			
Illiterate/semi-literate	5 (0.8)	1 (0.8)	0.95
Primary school	40 (6.5)	8 (6.1)	
Junior high school	462 (7.5.1)	103 (78.0)	
University or college	107 (17.4)	20 (15.2)	
Master and above	1 (0.2)	0 (0.0)	
**Employment**			
Farmer	143 (25.6)	23 (18.0)	0.07
Business	48 (8.6)	7 (5.5)	
Institution	13 (2.3)	6 (4.7)	
Others	354 (63.4)	92 (71.9)	
**Marital status**			
Unmarried	38 (5.9)	19 (13.8)	0.003
Married	603 (93.1)	119 (86.2)	
Divorced	7 (1.1)	0 (0.0)	
**History of APOs**			
Yes	465 (71.8)	43 (31.2)	0.56
No	183 (28.2)	95 (68.8)	
**History of syphilis infections**			
Yes	341 (52.6)	57 (41.3)	0.02
No	307 (47.4)	81 (58.7)	
**Period of maternal syphilis detection**			
Gestation	591 (91.2)	108 (78.3)	< 0.001
At delivery/postpartum	57 (8.8)	30 (21.7)	
**Mode of delivery**			
Cesarean section	331 (51.1)	75 (54.3)	0.55
Normal or assisted delivery	317 (48.9)	63 (45.7)	
**Number of childbirth**			
Singleton	636 (98.1)	131 (94.9)	0.053
Twins	12 (1.9)	7 (5.1)	
**Sex of the fetus**			
Male	347 (53.5)	60 (46.2)	0.15
Female	301 (46.5)	70 (53.8)	
**Pre-treatment**			
Positive	506 (79.4)	107 (80.5)	0.88
Negative	131 (20.6)	26 (19.5)	
**RPR/TRUST**			
Positive	259 (42.5)	47 (43.5)	0.87
Negative	350 (57.5)	60 (56.1)	
**TPPA**			
Positive	535 (98.5)	95 (95.0)	0.56
Negative	8 (1.5)	5 (5.0)	
**Newborns with preventive treatment**			
Yes	410 (63.3)	77 (59.2)	0.44
No	238 (36.7)	53 (40.8)	

Junior high school including junior high school, vocational high school, technical school, etc.

### 3.3. Prediction model analysis of APOs

Univariable logistic regression analysis was used on 20 variables to find significant predictors. It demonstrated that course, time of the first antenatal care, gestation week at syphilis diagnosis, and gestation age at delivery in weeks were potential independent predictors of APOs risk. Further multivariable analysis indicated that these four predictors are valid. The logistic regression results for these four predictors are shown in Table 3.

**Table 3:** Univariable and multivarible factors logistic regression analysis of APOs.

	**Univariable logistic regression analysis**				**Multivariable logistic regression analysis**			
	**OR**	**95% CI**		***P-*** **value**	**OR**	**95% CI**		***P-*** **value**
Course	0.572	0.439	0.744	< 0.001	0.695	0.487	0.992	0.04
Time of the first antenatal care	1.019	1.008	1.03	< 0.001	1.011	0.994	1.029	0.20
Gestation week at syphilis diagnosis	1.022	1	1.044	0.05	0.975	0.942	1.009	0.15
Gestation age at delivery in weeks	0.566	0.49	0.653	< 0.001	0.576	0.497	0.668	< 0.001

**Figure 1:**
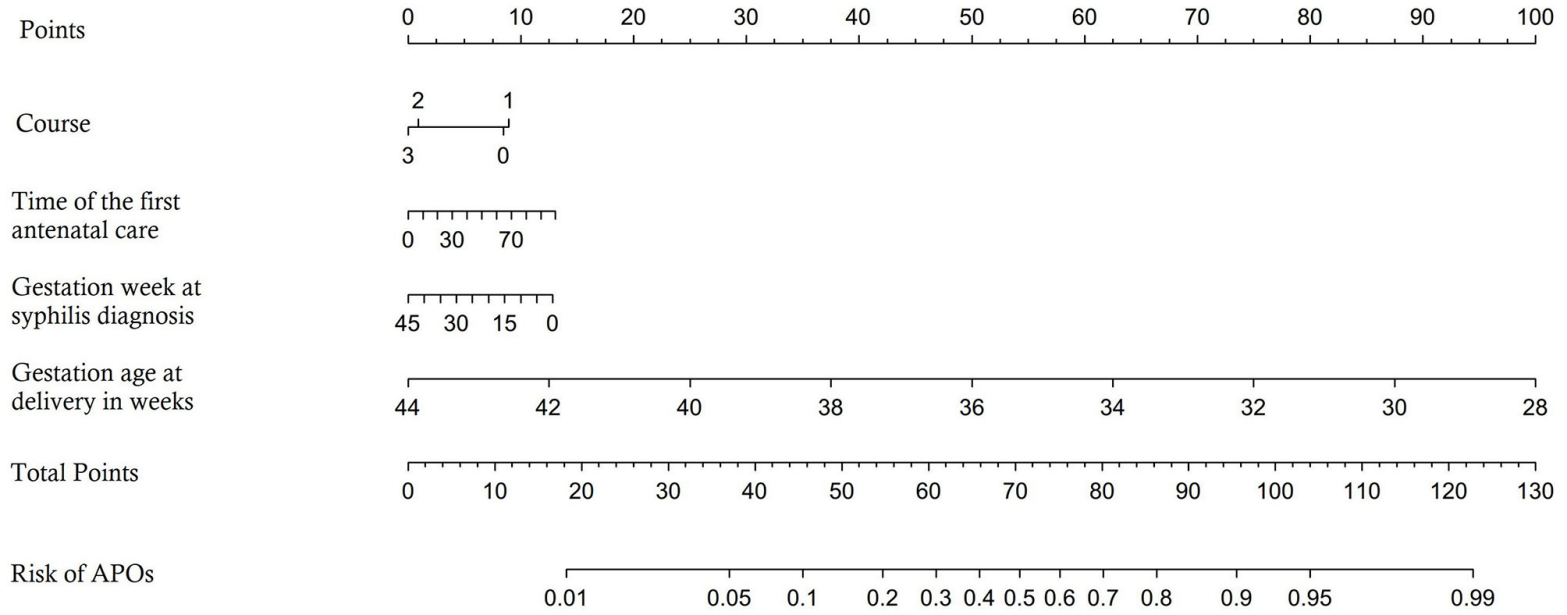
Nomogram prediction model for predicting the occurrence of APOs.

**Figure 2:**
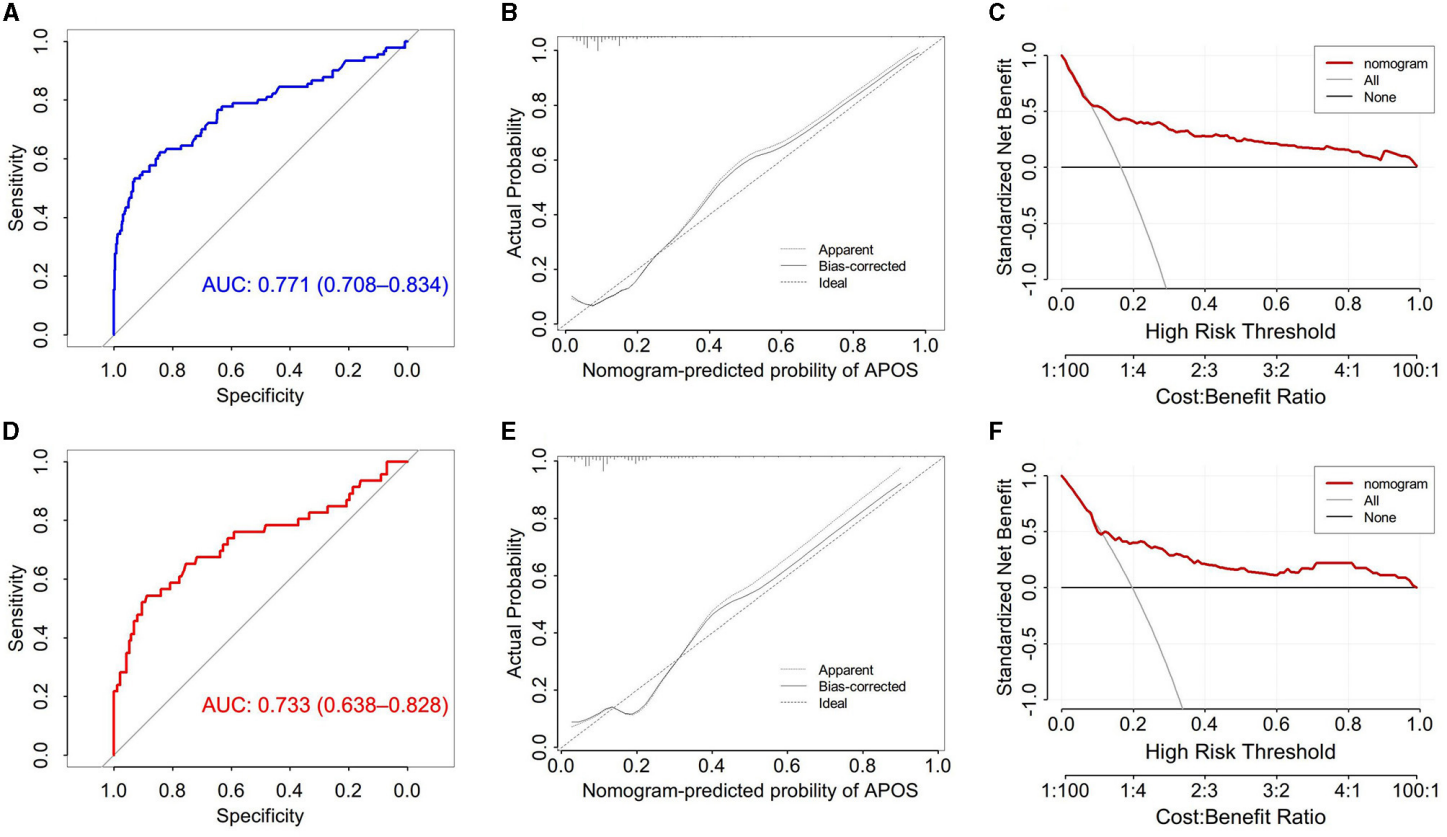
**(A)** Receiver operating characteristic curve for APOs prediction models in training set; **(B)** Calibration curve for the prediction model in the training set; **(C)** The decision curve of the model in the training set; **(D)** Receiver operating characteristic curve for APOs prediction models in testing set; **(E)** Calibration curve for the prediction model in the testing set; **(F)** The decision curve of the model in the testing set.

The predictors are applied to a model that is randomly divided into a training set and a testing set. Four independent predictors were used to establish a nomogram (Figure 1), and the points of each variable are shown in Table 4. The area under the curve (AUC) of our nomogram was 0.771 (95% CI, 0.708–0.834) in the training set (Figure 2A), showing good accuracy for predicting APOs. The calibration curves (Figure 2B) indicate a high degree of agreement between the predictions of the nomogram graphs and actual observations. Moreover, decision curve analysis (DCA; Figure 2C) demonstrated that using the nomogram to predict APOs performs well.

**Table 4:** Multivariable logistic regression models for prediction of APOs.

	**BETA**	**SE**	***Z***	**Estimate**	***P-*value**
Course	−0.36	0.18	−2.01	0.695 (0.487, 0.992)	0.04
Time of the first antenatal care	0.01	0.01	1.29	1.011 (0.994, 1.029)	0.20
Gestation week at syphilis diagnosis	−0.03	0.02	−1.44	0.975 (0.942, 1.009)	0.15
Gestation age at delivery in weeks	−0.55	0.08	−7.28	0.576 (0.497, 0.668)	< 0.001

The testing set included 235 pregnant women. Put the predictors into the testing set, and the model performs well. In the testing set, the AUC of the nomogram was 0.733 (95% CI, 0.708–0.834; Figure 2D), and the calibration curves (Figure 2E) showed good agreement between the predicted and actual probability of APOs occurrence. Additionally, DCA demonstrated that using the nomogram to predict APOs has a net benefit at all thresholds (Figure 2F).

## 4. Discussion

Our study showed that course, time to diagnosis, maternal clinical characteristics, marital status, and history of syphilis infection were associated with APOs. In the study of Zhang showed that a higher titer was a risk factor for APOs, and being married was a protective factor (22). Another study in Suzhou showed that with every two-fold increase in TPPA titer, the odds of congenital syphilis increase by 60% (23). Congenital syphilis presents with late abortion, intrauterine fetal death, stillbirth and low birth weight depending on the severity. Early manifestations of syphilis in the neonatal period include aseptic meningitis, seizures, rash, and neonatal death (24). Maternal age < 25 years and maternal mental health status are risk factors for congenital syphilis, as these can be barriers to accessing primary and prenatal care (25). But this risk can be reduced by timely and adequate treatment. Unlike other studies (26), age was not a risk factor for APOs, presumably related to sample size.

Based on four predictors, our model was established as a tool that can predict the APOs risk of syphilis-infected women. We found that pregnant women with syphilis who had adverse pregnancy outcomes had fewer treatment sessions. Similar results have been described in previous studies, and inadequate treatment and treatment initiated in late pregnancy remained risk factors for APOs (4, 26, 27). A Retrospective Cohort Study in China compared different treatments and concluded that China-recommended therapy, two courses of penicillin treatment, might have a large in reducing multiple APOs (28). If maternal treatment during pregnancy is inadequate, close evaluation and empirical treatment of the infant needs to be considered.

The time of the first antenatal care and gestation week at syphilis diagnosis was also associated with pregnancy outcomes, consistent with other studies (6, 29). Early antenatal care can facilitate early detection of maternal syphilis and prompt treatment for those with positive tests (30). Estimated by a hazards model: each 1-week delay in the start of prenatal care may increase the risk of congenital syphilis by ~10% (31). Factors that contribute to the occurrence of APOs are related to the gestational age at the time of diagnosis and may be related to the management received after diagnosis. A multivariable logistic regression showed that syphilis-infected mothers diagnosed at >36 weeks' gestational age were ~25 times more likely to deliver an infant with congenital syphilis compared to women diagnosed at ≤ 12 weeks' gestational age (23). A massive health education program in Brazil has improved the quality of maternal syphilis and reduced the incidence of cs. quality of treatment of CS (32). Therefore, health education and promotion of sexually transmitted diseases such as syphilis should be conducted at premarital checkups or earlier.

The full gestational age at delivery in weeks is a crucial predictor of the occurrence of APOs. This factor was hardly discussed in the same type of studies. A cohort study in Norway (33) showed that the risk of APOs was highest at 37 and 42 weeks and lowest at 39 weeks. Consistent with our findings.

A review evaluating the optimal timing of prenatal interventions to prevent mother-to-child transmission of syphilis and APOs suggests that women who received interventions (including screening and treatment) in the first and second trimesters of pregnancy have a lower incidence of adverse outcomes compared to those in the late stages of the pregnancy (19). The risk of trans-placental infection is 60%−80% and the likelihood of infection increases in the second half of pregnancy (17). Infants of women who first sought antenatal care in the third trimester or at delivery often already have congenital syphilis (34). In China, all pregnant women could receive two courses of treatment for free as soon as a diagnosis of syphilis infection was made during pregnancy (35). ACOG also recommends that screening for sexually transmitted infections, including HIV, syphilis and hepatitis, should be offered to women at high risk for these infections as part of their pregnancy care. Future efforts should include health promotion and improving treatment adherence. It is recommended to start prenatal care as early as possible, which means syphilis can be diagnosed and treated as soon as possible.

We analyzed the risk factors for adverse outcomes in pregnant women with syphilis and based on this made a model that could quantify the risk. Based on the estimated risk results, obstetricians can provide treatment recommendations to their patients. Our study is helpful to take effective intervention measures for syphilis mothers and newborns in time, so as to significantly reduce the incidence of APOs, which has significant clinical significance in ensuring maternal and infant health and improving pregnancy outcome.

Our study has the following limitations. First, we lack the support of other databases for external validation of model accuracy. Second, only women who delivered ≥28 gestational weeks were included in the study. Hence, some adverse birth outcomes, including early fetal loss and miscarriage, might be underestimated. In a follow-up study, we plan to add comparisons with other data and consider using more accurate artificial intelligence models to predict APOs to refine our model.

## 5. Conclusions

In summary, this study identified risk factors for the occurrence of adverse pregnancy outcomes in pregnant women with syphilis and used them to develop a predictive model. Our study complements the results of the study of pregnant women with syphilis and will help health workers to identify and assist those at risk.

## Data availability statement

The original contributions presented in the study are included in the article/Supplementary material, further inquiries can be directed to the corresponding author: Y-NM, ma_yana@163.com.

## Ethics statement

This study was approved by the Ethics Committee of The Affiliated Infectious Diseases Hospital of Soochow University (No. 2019002). The studies were conducted in accordance with the local legislation and institutional requirements. The participants provided their written informed consent to participate in this study. Written informed consent was obtained from the individual(s) for the publication of any potentially identifiable images or data included in this article.

## Author contributions

Y-WZ, M-YL, X-HY, XL, and Y-NM made substantial contributions to the design of the work. Y-WZ and M-YL analyzed the data and drafted the manuscript. X-YH collected the data. X-HY and X-YH designed the research. X-HY and WS analyzed the data. XL and Y-NM performed the research. All authors have read and approved the final manuscript.
